# The Diabetes Location, Environmental Attributes, and Disparities Network: Protocol for Nested Case Control and Cohort Studies, Rationale, and Baseline Characteristics

**DOI:** 10.2196/21377

**Published:** 2020-10-19

**Authors:** Annemarie G Hirsch, April P Carson, Nora L Lee, Tara McAlexander, Carla Mercado, Karen Siegel, Nyesha C Black, Brian Elbel, D Leann Long, Priscilla Lopez, Leslie A McClure, Melissa N Poulsen, Brian S Schwartz, Lorna E Thorpe

**Affiliations:** 1 Department of Population Health Sciences Geisinger Danville, PA United States; 2 Department of Epidemiology University of Alabama at Birmingham School of Public Health Birmingham, AL United States; 3 Department of Epidemiology and Biostatistics Drexel University Dornsife School of Public Health Philadelphia, PA United States; 4 Centers for Disease Control and Prevention Atlanta, PA United States; 5 Noire Analytics, LLC Birmingham, AL United States; 6 Department of Population Health NYU Langone Health New York, NY United States; 7 Department of Biostatistics University of Alabama at Birmingham School of Public Health Birmingham, AL United States; 8 Department of Environmental Health and Engineering Johns Hopkins Bloomberg School of Public Health Baltimore, MD United States

**Keywords:** type 2 diabetes, built environment, social environment, disparities

## Abstract

**Background:**

Diabetes prevalence and incidence vary by neighborhood socioeconomic environment (NSEE) and geographic region in the United States. Identifying modifiable community factors driving type 2 diabetes disparities is essential to inform policy interventions that reduce the risk of type 2 diabetes.

**Objective:**

This paper aims to describe the Diabetes Location, Environmental Attributes, and Disparities (LEAD) Network, a group funded by the Centers for Disease Control and Prevention to apply harmonized epidemiologic approaches across unique and geographically expansive data to identify community factors that contribute to type 2 diabetes risk.

**Methods:**

The Diabetes LEAD Network is a collaboration of 3 study sites and a data coordinating center (Drexel University). The Geisinger and Johns Hopkins University study population includes 578,485 individuals receiving primary care at Geisinger, a health system serving a population representative of 37 counties in Pennsylvania. The New York University School of Medicine study population is a baseline cohort of 6,082,146 veterans who do not have diabetes and are receiving primary care through Veterans Affairs from every US county. The University of Alabama at Birmingham study population includes 11,199 participants who did not have diabetes at baseline from the Reasons for Geographic and Racial Differences in Stroke (REGARDS) study, a cohort study with oversampling of participants from the Stroke Belt region.

**Results:**

The Network has established a shared set of aims: evaluate mediation of the association of the NSEE with type 2 diabetes onset, evaluate effect modification of the association of NSEE with type 2 diabetes onset, assess the differential item functioning of community measures by geographic region and community type, and evaluate the impact of the spatial scale used to measure community factors. The Network has developed standardized approaches for measurement.

**Conclusions:**

The Network will provide insight into the community factors driving geographical disparities in type 2 diabetes risk and disseminate findings to stakeholders, providing guidance on policies to ameliorate geographic disparities in type 2 diabetes in the United States.

**International Registered Report Identifier (IRRID):**

DERR1-10.2196/21377

## Introduction

### Background

An estimated 10.5% of the US population has diabetes, and these 34 million individuals are at an increased risk for coronary artery disease, cerebrovascular disease, and other complications [1,2]. Approximately 90% to 95% of people with diabetes have type 2 diabetes (T2D) [1]. Another 88 million individuals have prediabetes, defined as having elevated glucose levels above normal but below the threshold for diabetes, and are at elevated risk of developing T2D [3,4]. Diabetes prevalence and incidence vary substantially by geographic region [5-7]. In 2013, there was a six-fold difference between counties with the lowest and highest diabetes prevalence [6]. A large body of literature links community and environmental factors (hereafter referred to as community factors) to T2D and obesity, one of the risk factors for T2D [8-15]; however, the mechanisms for these links remain poorly understood. Moreover, there are inconsistencies in this body of literature. Identifying the community factors driving T2D disparities and the pathways through which these factors influence T2D is essential to informing geographically targeted policy interventions that reduce the risk of T2D and related outcomes.

Researchers have identified consistent associations of community-level socioeconomic factors (eg, community poverty rate) with T2D prevalence and incidence. However, findings related to aspects of the built (eg, food and physical activity establishment environment, land use environment) and natural environment (eg, greenness) and T2D risk have been less uniform [9-19]. This may be due, in part, to methodologic variations in measuring community factors, including differences in spatial scales, data sources, and measurement approaches [20].

Among the challenges to creating a cohesive body of research is a lack of consistent approaches to conceptualizing and operationalizing the geographic area in which community factors are thought to be relevant to health [19-22]. Furthermore, the size and boundaries of spatial scales most relevant to health may vary according to community type (eg, across the gradient from urban to rural). Community type is also an important consideration in measurement development, as measurement of the same community factors may require different approaches [23]. For example, car ownership may be a basic necessity for individuals living in rural areas but more of a luxury for individuals living in urban areas with good public transportation options. Thus, car ownership may work differently as an indicator of the neighborhood socioeconomic environment (NSEE) in urban versus rural areas [24]. This differential item functioning may contribute to inconsistencies observed in the literature as the same measure (eg, proportion who own a car) could hold different meanings in different community types.

Community and health research is also vulnerable to structural confounding, which occurs when individual and contextual factors strongly predict residence in a certain community [25,26]. In the presence of structural confounding, certain measures, owing to social sorting, are largely nonoverlapping, resulting in an inability to examine their independent influences on the outcome of interest. For example, in some settings, the distribution of persons across categories of the NSEE and racial residential segregation may reveal a lack of comparable groups across key strata, resulting in analytical challenges of nonpositivity that prohibit causal contrasts across levels of exposure [26]. Finally, capturing the complex interactions among multiple community factors on health can be challenging [27]. As a result, previous research has largely evaluated co-occurring community factors in isolation.

### Objectives

The increasing availability of longitudinal, individual-level data from electronic health record (EHR) networks [28] and cohort studies, coupled with advances in geographic information systems (GISs), provides new opportunities to examine the effects of community factors on health. In 2017, the Diabetes LEAD (Location, Environmental Attributes, and Disparities) Network was established to identify the contributions of modifiable community factors on T2D risk. The Network includes researchers from 4 academic institutions who collaborate to address the methodological challenges previously described to investigate a range of community factors across the United States. The Network aims to guide policy decision making to reduce the burden of T2D across the United States. This paper aims to describe the Diabetes LEAD Network, its study populations, and the methodologies used to investigate the community factors that are associated with T2D onset and related outcomes.

## Methods

### Network Overview

**Figure 1:**
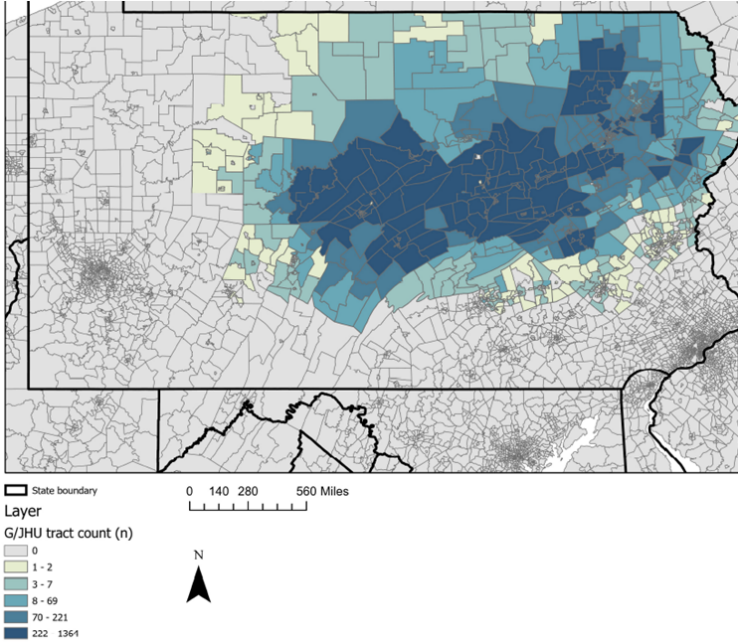
Geographic coverage of Pennsylvania in the Geisinger and Johns Hopkins University study population: participants in each site by census tract. G/JHU: Geisinger and Johns Hopkins University.

**Figure 2:**
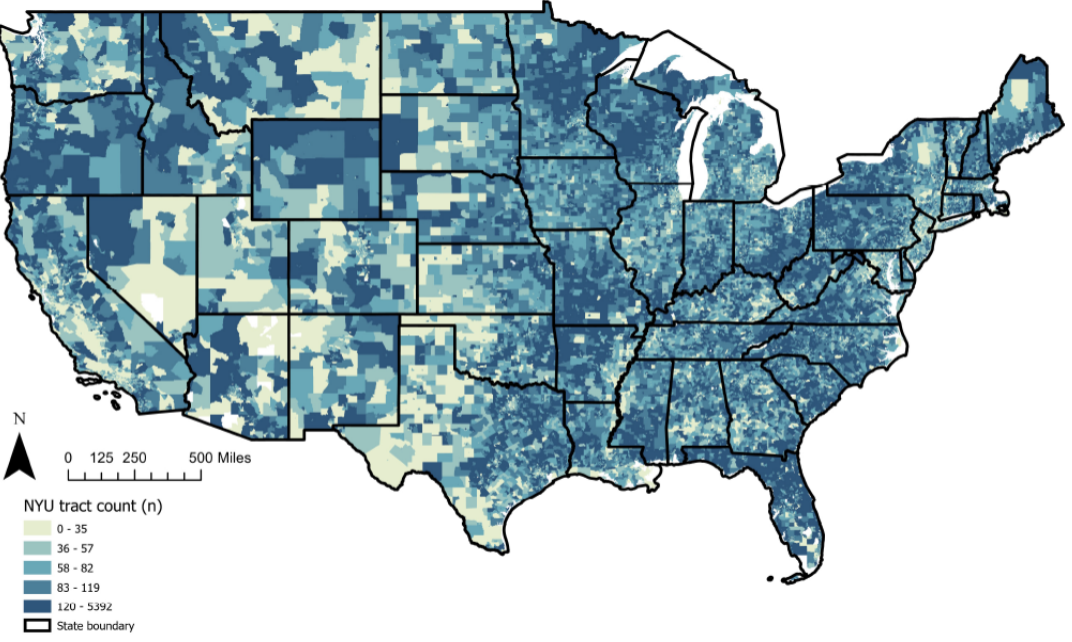
Geographic coverage of the New York University study population: participants in each site by census tract. NYU: New York University.

**Figure 3:**
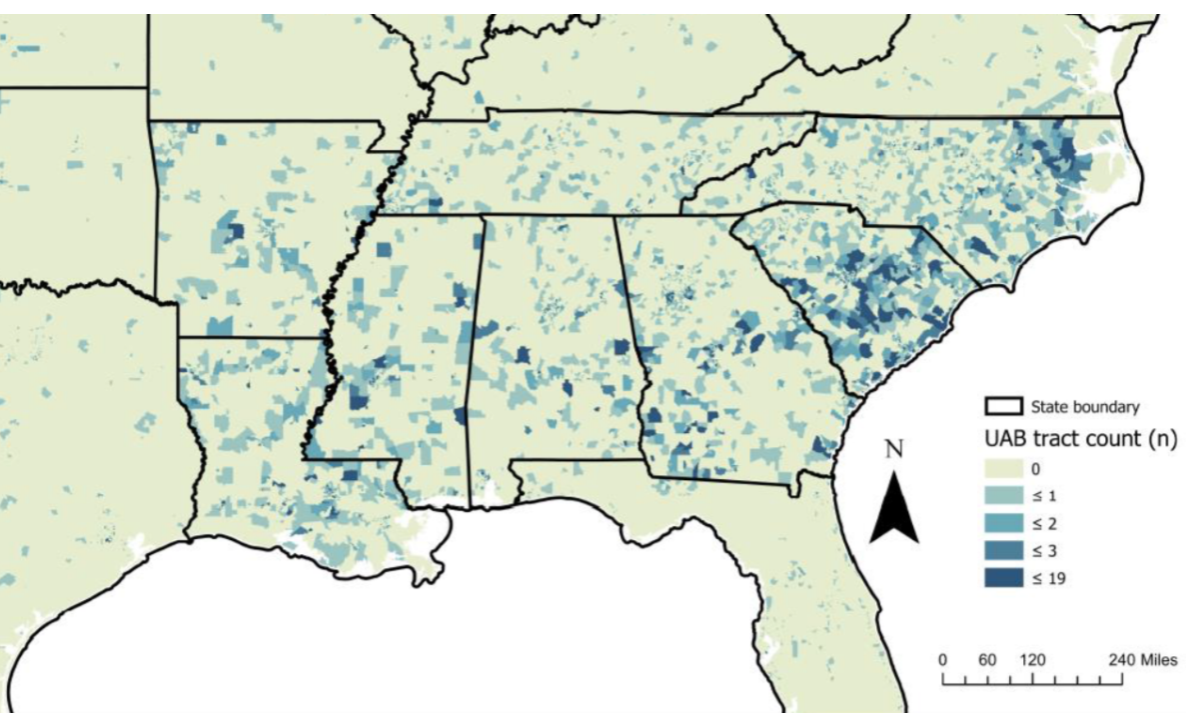
Geographic coverage of the University of Alabama at Birmingham study population in the Stroke Belt region and surrounding states: participants in each site by census tract. Participants are from all 48 contiguous states. The map reflects the Stroke Belt region and surrounding states. UAB: University of Alabama at Birmingham.

The Diabetes LEAD Network is a research collaboration of 4 academic centers: Drexel University, Geisinger and Johns Hopkins University (G/JHU), New York University School of Medicine (NYU), and the University of Alabama at Birmingham (UAB). The Centers for Disease Control and Prevention (CDC) funded the Network to bring together institutions with diverse but complementary expertise and a rich array of data assets. Three study sites—G/JHU, NYU, and UAB—use longitudinal data, such as EHRs, administrative claims, and survey data on distinct populations and geographies in the United States (Tables 1-3; Figures 1-3). Drexel, the data coordinating center (DCC), is leading the development of a set of harmonized community factors, health outcomes, and analysis plans (Tables 4 and 5) that will be applied to each study site’s cohort and geography. Each site has its own set of study aims that examine community factors and T2D outcomes, including T2D onset, obesity, and other cardiometabolic conditions. Working collaboratively, the study sites and the CDC also developed a shared set of aims that complement site-specific aims (Textbox 1). We first describe the shared Network aims and then describe the site-specific aims.

**Table 1:** Diabetes Location, Environmental Attributes, and Disparities Network study site populations for Network-specific aims.

Study site, G/JHU^a^ (n=578,485^b^)		NYU^c^ (n=6,082,246)	UAB^d^ (n=11,199)
**Study design**			
	Nested case control and cohort	Cohort^e^	Cohort
**Source population**			
	All Geisinger patients (N=1,605,922)	All patients in the Veterans Affairs EHR^f^ with at least one primary care visit since 1999 (N=8,346,280)	REGARDS^g^ participants at baseline (2003-2007; N=30,239)
**Exclusion criteria (sample size excluded)**			
	Patients with <2 primary care visits^h^ (January 1, 2006 - December 31, 2016; n=970,785)	Patients with <2 primary care visits at least 30 days apart during the 5 years (before January 1, 2008; n=4,270,462)	Participants without a second visit (2013-2016; n=14,089)
	Patients with a residential address outside of one of the 37 counties in the Geisinger primary service area (n=56,652)	Patients with diabetes^i^ before or on January 1, 2008 (n=1,049,423)	Participants with diabetes^i^ at baseline (n=2729) or missing diabetes status at baseline (n=521; combined, n=3250)
	N/A^j^	N/A	Participants missing diabetes status at second visit (n=1580)
	N/A	N/A	Unable to assign census tract using RECVD^k^ data set (n=121)

^a^G/JHU: Geisinger and Johns Hopkins University.

^b^Study population will vary based on study design (ie, nested case control or cohort).

^c^NYU: New York University.

^d^UAB: University of Alabama at Birmingham.

^e^These numbers reflect only those entering the cohort on inception date (January 1, 2008). The dynamic cohort allowed patients to enter the cohort through December 31, 2016 (n=3,113,391). Total cohort population was 6,082,246.

^f^EHR: electronic health record.

^g^REGARDS: Reasons for Geographic and Racial Differences in Stroke study.

^h^Includes internal medicine, family medicine, pediatrics, and obstetrics or gynecology.

^i^See Table 5 for type 2 diabetes definitions.

^j^N/A: not applicable.

^k^RECVD: Retail Environment and Cardiovascular Disease.

**Table 2:** Location, Environmental Attributes, and Disparities Network study population characteristics by site.

Characteristics		G/JHU^a^	NYU^b^	UAB^c^
Study population, n		578,485	6,082,246	11,199
**Sex, n (%)**				
	Male	254,218 (43.94)	5,578,056 (91.71)	4946 (44.16)
	Female	324,267 (56.05)	504,020 (8.28)	6253 (55.83)
**Age (years), n (%)^d^**				
	<18	132,341 (22.87)	0 (0)	0 (0)
	18-29	92,458 (15.98)	450,504 (7.40)	0 (0)
	30-39	67,185 (11.61)	550,910 (9.05)	0 (0)
	40-49	67,996 (11.75)	753,811 (12.39)	649 (5.79)
	50-59	74,641 (12.90)	1,168,452 (19.21)	3445 (30.76)
	60-69	63,946 (11.05)	1,566,257 (25.75)	4547 (40.60)
	≥70	79,918 (13.81)	1,592,179 (26.17)	2558 (22.84)
**Race, n (%)**				
	Black	23,302 (4.02)	920,596 (15.13)	3672 (32.78)
	American Indian or Alaska Native	655 (0.11)	56,928 (0.93)	0 (0)
	Asian	4616 (0.79)	58,555 (0.96)	0 (0)
	Native Hawaiian or other Pacific Islander	2746 (0.47)	60,441 (0.99)	0 (0)
	White	542,128 (93.71)	4,411,233 (72.52)	7527 (67.21)
**Ethnicity, n (%)**				
	Hispanic	25,274 (4.36)	331,376 (5.44)	0 (0)
	Non-Hispanic	553,211 (95.63)	5,750,870 (94.55)	11,199 (100.00)
**Setting of residential address, n (%)**				
	Higher density urbanized area	48,374 (8.36)	688,488 (11.31)	1809 (16.15)
	Lower density urbanized area	76,301 (13.18)	2,139,912 (35.18)	4524 (40.39)
	Suburban and small town (UC)^e^	178,548 (30.86)	1,328,278 (21.83)	2644 (23.60)
	Rural	275, 272 (47.58)	1,781,743 (29.29)	2222 (19.84)
Diagnosed with type 2 diabetes by the end of the follow-up period, n (%)^f^		64,214 (11.10)	936,627 (15.39)	1408 (12.57)

^a^G/JHU: Geisinger and Johns Hopkins University.

^b^NYU: New York University.

^c^UAB: University of Alabama at Birmingham.

^d^G/JHU: age as of date of the data pull (2016); NYU: age calculated by subtracting year of birth from cohort entry year; UAB: age at baseline.

^e^UC: urban cluster

^f^See Table 5 for diabetes definitions. G/JHU: 2008 to 2016; NYU: 2008 to 2016; UAB: 2003 to 2016.

**Table 3:** Location, Environmental Attributes, and Disparities Network individual-level data elements available by study site for Network aims.

Data elements		G/JHU^a,b^	NYU^c^	UAB^d,e^
**Individual-level data**				
	Demographic data (yes/no)	Yes	Yes	Yes
	Residential address data	Most recent only	Longitudinal	Longitudinal
	Socioeconomic data	Longitudinal	Longitudinal	Longitudinal
**Health-related data**				
	BMI	Longitudinal	Longitudinal	Longitudinal
	Vital signs (eg, blood pressure)	Longitudinal	Longitudinal	Longitudinal
	Diagnoses	Longitudinal	Longitudinal	Longitudinal
	Treatment	Longitudinal	Longitudinal	Longitudinal^f^
	Biomarkers (type)	EHR^g^ laboratory data	EHR laboratory data	HbA_1c_^h^ available on subset only^i^

^a^G/JHU: Geisinger and Johns Hopkins University.

^b^Includes only data in the EHR-based study. See Multimedia Appendix 1 for additional data collected in the primary data collection study.

^c^NYU: New York University School of Medicine.

^d^UAB: University of Alabama at Birmingham.

^e^Longitudinal data are available at 2 time points—baseline (2003-2007) and second in-home exam (2013-2016).

^f^Adjudicated (confirmed by review of medical records) coronary heart disease, stroke, end-stage renal disease, and death available throughout follow-up.

^g^EHR: electronic health record.

^h^HbA_1c_: glycated hemoglobin.

^i^n=2694 at baseline and n=2527 at follow-up examination.

**Table 4:** Community and environmental domains for Network-wide aims.

Domain	Data source and years	Spatial scale	Description
Neighborhood socioeconomic environment	US Decennial Census and American Community Survey (5-year estimates; 2000, 2010, 2006-2010, and 2008-2012)	Census tract	Area-level index derived from a z-score sum of indicators of the community’s social and economic characteristics [29]: percentage of males and females with less than a high school education, percentage of males and females unemployed, percentage of households earning less than US $30,000 per year, percentage of households in poverty, percentage of households on public assistance, and percentage of households with no cars
Food establishment environment	RECVD^a^ Geocoded Business Level Data set, derived from NETS^b^ (1997-2014)	Network buffer around the population-weighted centroid of census tracts	Area-level absolute and relative measures: density of supermarkets (including medium-sized grocers) and fast food restaurants (per square kilometer), ratio of supermarkets to all food stores, and ratio of fast food restaurants to all restaurants and eating places
Physical fitness establishment environment	RECVD Geocoded Business Level Data set, derived from NETS^b^ (1997-2014)	Network buffer around the population-weighted centroid of census tracts	Area-level density of physical activity venues per square kilometer (eg, gyms, membership sports and recreation clubs, athletic organizations)
Land use environment	RECVD National Land Cover Database, ESRI StreetMap, RECVD NETS, US Decennial Census (2006, 2009, and 2010)	Census tract	Area-level index derived from a z-score sum of 7 indicators: average block length, average block size, intersection density, street connectivity, density of walkable establishments per square mile, percent developed land, and household density per square mile
Leisure-time physical activity environment	CDC’s^c^ Division of Population Health, National Center for Chronic Disease Prevention and Health Promotion. Derived from the Homeland Security Infrastructure Program Gold 2011 database^d^, Environmental Systems Research Institute Arc Geographic Information System (ESRI ArcGIS) 10.1 Data DVD 2010	Census tract	Spatial access to parks measured by population-weighted distance to the 7 closest parks from the census tract’s population-weighted centroid [30,31]

^a^Drexel University Urban Health Collaborative. The Retail Environment and Cardiovascular Disease (RECVD) Project.

^b^National Establishment Time-Series (NETS) database.

^c^CDC: Centers for Disease Control and Prevention.

^d^The Homeland Infrastructure Foundation-level Data Working Group NAVTEQ from Homeland Security Infrastructure Program Gold 2011 Database.

**Table 5:** Definition of type 2 diabetes.

Study site	Diagnoses codes	Laboratory measures^a^	Medication orders^b^	Exclusions
Geisinger and Johns Hopkins University	ICD^c^-9, ICD-10, or EDG^d^ code for T2D on two separate dates	≥1 elevated HbA_1c_^e^ or glucose measure and ≥1 diagnosis code for T2D	≥1 T2D medication order	≥10 years of T1D^f^ diagnoses and <5 years of T2D^g^ diagnoses or first diabetes code before 10 years of age or only meet criteria during pregnancy
New York University School of Medicine	ICD-9 or ICD-10 code for T2D on two separate dates	≥2 elevated HbA_1c_ or glucose measure and ≥1 diagnosis code for T2D	≥1 T2D medication order	N/A^h^
University of Alabama at Birmingham^i^	N/A	Elevated glucose measure at study visit	Self-report of T2D medication	N/A

^a^HbA_1c_≥6.5%, random glucose≥200 mg/dl, and fasting glucose≥126 mg/dl.

^b^Excluding Metformin and Acarbose: Reasons for Geographic and Racial Differences in Stroke project.

^c^ICD: International Classification of Diseases.

^d^EDG: Epic Diagnostic code Groupers.

^e^HbA_1c_: glycated hemoglobin.

^f^T1D: type 1 diabetes.

^g^T2D: type 2 diabetes.

^h^N/A: not applicable.

^i^From the Reasons for Geographic and Racial Differences in Stroke Study.

**Site-specific aims.**

Geisinger and Johns Hopkins University:

- To evaluate associations of chronic environmental contamination [30] (eg, abandoned coal mine lands); the food environment; the physical activity environment; land use environment, the natural environment (eg, greenness); community type (eg, urban/rural); and community socioeconomic deprivation (CSD) with type 2 diabetes (T2D) onset and control and coronary heart disease (CHD) onset within communities.
- To evaluate mediating pathways (eg, food, physical activity environment) between the neighborhood socioeconomic environment and T2D onset (through LEAD Network Aim 1).
- To evaluate mediating pathways (eg, stress, health behaviors) between community factors and T2D control among 1000 individuals with T2D living in 40 communities.
- To evaluate potential effect modification by key individual (eg, age, Medical Assistance) and community factors (eg, CSD) of relations between community factors and T2D and CHD within communities.

New York University School of Medicine:

- Using public-use data sources, determine independent and joint association between novel community measures and county-level prevalence of outcomes (diabetes, obesity, and diabetes-obesity prevalence discordance profile), controlling for other county measures (eg, population density, socioeconomic status, and demographic distributions).
- Measure the impact of modifiable community characteristics such as food and housing environments on (a) risk of a new T2D diagnosis or (b) being obese (BMI≥30 kg/m^2^) in a large cohort of Veterans Affairs patients, adjusting for community and individual-level covariates in multilevel regression models.
- Use mediation analysis to examine mediating pathways between modifiable community contexts and T2D.

University of Alabama at Birmingham:

- To determine the association of community-level social determinants of health with the prevalence and incidence of T2D and hypertension, separately.
- To determine if pharmacologic treatment patterns and hospitalization rates vary by community-level social determinants of health for those with T2D and hypertension, separately.
- To determine if awareness and treatment of T2D and risk of cardiovascular complications varies by community-level and individual-level social determinants of health.

### Network Aims

**Figure 4:**
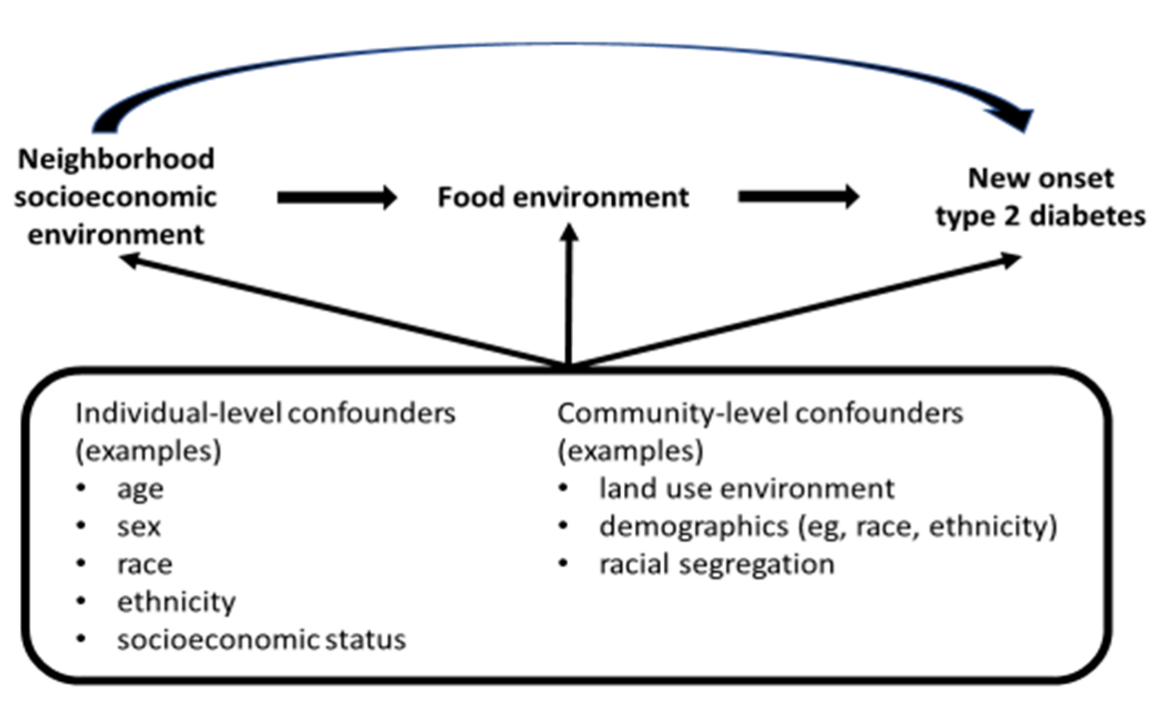
Conceptual framework for mediation of the association between neighborhood socioeconomic environment and type 2 diabetes: food environment as an example mediator.

The Network aims to evaluate the association of community factors and T2D outcomes (aims 1 and 2) and to evaluate and address the previously described methodological challenges of community and health research (aims 3 and 4):

1. Evaluate the mediation of the association of NSEE with new-onset T2D. This aim reflects a conceptual framework (Figure 4) that proposes that NSEE influences T2D onset through other community pathways, including the food, physical activity (fitness and leisure) environments, and exposure to fine particulate matter (≤2.5 µ, particulate matter_2.5_).
2. Define and test effect modifiers (eg, age, sex, race) of the association of NSEE with new-onset T2D.
3. Assess the differential item functioning of community measures by geographic region and community type.
4. Evaluate the impact of the spatial scale used to measure community factors (eg, buffer, census tract, county) on associations with new-onset T2D.

### Network Populations and Geographic Coverage

The Diabetes LEAD Network draws from individuals living in all 50 US states (Figures 1-3). The G/JHU participants were selected from among 1.6 million individuals in the Geisinger EHR, spanning 37 counties in central and northeastern Pennsylvania (Table 1). The G/JHU participants range in age from 10 to 97 years, are predominately White (542,128/578,458, 93.71%) and non-Hispanic (553,211/578,458, 95.66%), reflecting the region’s population [32]. More than 40% of G/JHU participants reside in areas that the US Census Bureau categorizes as rural. The NYU population of veterans spans all US counties. NYU assembled a baseline cohort of veterans who do not have diabetes (n=6,082,246) and are receiving primary care through Veterans Affairs. Veterans are mostly male (5,578,056/6,082,246, 91.71%) and predominantly White (4,411,233/6,082,246, 72.53%) followed by Black (920,596/6,082,246, 15.14%).

These demographic groups are represented by large sample sizes. The UAB population includes participants from the Reasons for Geographic and Racial Differences in Stroke (REGARDS) study cohort [33]. At baseline (2003-2007), the REGARDS study enrolled 30,239 non-Hispanic Black and non-Hispanic White adults aged 45 years and older with oversampling of participants from the Stroke Belt region (Alabama, Arkansas, Georgia, Louisiana, Mississippi, North Carolina, South Carolina, and Tennessee) [34]. To assess T2D onset, participants without T2D at baseline and who completed the follow-up in-home examination (n=11,199) will be evaluated. Patients were not invited to comment on the cohort development or study design.

### Network Data Sources and Measurement

The DCC is leading the development of harmonized, Network-wide approaches to measuring community factors of interest and T2D outcomes. To develop measures of community factors (Table 4), the DCC is using archival data available at the national level, including publicly available data (eg, US Census) and data elements previously created for the Retail Environment and Cardiovascular Disease (RECVD) study. The RECVD study has longitudinal measures of food, fitness, and social establishments based on the National Establishment Time Series (NETS), a data source that includes information on more than 58 million US business establishments from 1990 to 2014. For each community factor, the DCC has partnered with a study site with relevant expertise to make decisions regarding data sources, spatial scale, exposure assignment, and approach to measurement.

The DCC is applying a range of measurement techniques to define community factors, including data reduction and measurement models. Measurement development is stratified by community type at the census tract level using a modification of the Rural-Urban Commuting Area (RUCA) from the US Department of Agriculture developed by the Network [35]. After collapsing the original 10 RUCA categories into 3, the DCC further divided census tracts within urbanized areas into 2 categories based on land area, resulting in 4 community-type categories that reflect distinct typologies along the rural-urban continuum.

To the extent possible, the Network is harmonizing approaches to measure T2D onset (Table 5) and diabetes-related outcomes. G/JHU and NYU have worked together to develop EHR-based algorithms based on their previous work [36,37] and diagnosis criteria from the American Diabetes Association [38], using a combination of diagnosis codes, medications, and laboratory measures. With coordination from the DCC, the sites are also standardizing approaches to measure potential confounders, mediators, and effect modifiers.

### Network Analyses

For each Network-wide aim, the study sites will conduct analyses among their study populations based on a common analytic plan. The DCC is coordinating the development of the analytic plan, harmonizing analytical approaches, including the selection of confounding, mediating, and modifying variables of interest, model building, and model diagnostics. Sites will conduct site-specific sensitivity analyses that include relevant data elements that may not be available Network-wide. This approach allows us to examine consistency in results while leveraging the unique data available at individual sites.

For aims 1 and 2, the Network will employ methods to account for group-level and individual-level data, including multilevel models, Bayesian approaches, and generalized estimating equation models. The Network will conduct causal mediation analysis for aim 1 [39]. For aim 2, we will evaluate effect modification through the inference of interaction terms, creating cross-products between our contextual domains of interest and a predetermined set of individual- and community-level variables, such as age, sex, and race. To guide model development for these aims, sites are developing causal diagrams to formulate and test theoretically based pathways, identify potential confounding influences, and account for potential interaction between measures (Figure 4) [40,41]. To assess spatial residual autocorrelation, the Network will calculate I statistics by Moran (local and global) [42] and use modeling approaches that account for spatial residual autocorrelation, if needed. The Network will conduct sensitivity analyses to evaluate how approaches to measurement of outcomes and community factors impact observed associations.

For aim 3, the Network is exploring strategies to evaluate and address nonpositivity, including propensity scores [43], stratification by community type for all analyses, and latent profile analysis to evaluate community typology [44]. For aim 4, we are evaluating community factors at multiple spatial scales (eg, census tract, network buffer around population-centroid). We will compare the associations of community factors measured at different scales with T2D to assess the influence of the modifiable areal unit problem [21]. The study sites also have site-specific aims, as described below.

### Site Descriptions

#### Geisinger and Johns Hopkins University

The Environmental Health Institute, a joint collaboration between Geisinger, Johns Hopkins Bloomberg School of Public Health, and Johns Hopkins School of Medicine, is evaluating the influence of community factors on T2D onset and control and cardiometabolic outcomes in Pennsylvania (Textbox 1) using a combination of primary and secondary data collection. The team is conducting the study among patients from Geisinger, a health system serving 1.6 million patients in central and northeastern Pennsylvania. To be eligible for study, individuals had to reside in one of 37 counties in Geisinger’s service area and have at least two Geisinger primary care visits from 2006 to 2016 (Table 1). The Geisinger primary care patient population represents the age, sex, and racial and ethnic distribution of the general population of the region [32]. The region’s population is residentially stable, with an annual out-migration rate of approximately 1% in all but two counties according to US Census Bureau data.

G/JHU is evaluating the main effects of 8 community factors: NSEE, food environment, fitness environment, leisure-time physical activity environment, land use environment, greenness, blue space (aquatic environments such as coasts, lakes, and rivers), and chronic environmental contamination [45]. G/JHU has previously reported associations between these factors and obesity and glycated hemoglobin (HbA_1c_) [46-51]. G/JHU is using data from publicly available sources (eg, US Census, American Community Survey [ACS], Moderate Resolution Imaging Spectroradiometer from the National Aeronautics and Space Administration’s Terra satellite, Pennsylvania Department of Transportation, TeleAtlas) and commercial data to generate measures for these factors. For the Network aims, the team is working with the DCC to guide decisions on the land use and physical fitness environment measures.

For site-specific aims, G/JHU outcomes include T2D onset, T2D control, and cardiometabolic outcomes (Textbox 1). G/JHU is conducting 2 types of studies to evaluate the associations between community factors and T2D outcomes and mediation and moderation of these associations. EHR-based analyses will be used for both Network- and site-specific aims. A primary data collection study will be used for additional site-specific aims, as much of the data collected in this study will be uniquely available at the G/JHU site (Multimedia Appendix 1).

G/JHU is using a mix of nested case-control and retrospective cohort study designs to achieve site-specific aims, using logistic and linear regression as appropriate. To account for correlation due to both place and space, G/JHU is using generalized estimating equations and multilevel modeling. To examine mediators of the association between NSEE and T2D onset (Network Aim 1), Geisinger will apply a nested case-control design and formal mediation models that include T2D onset cases (n=15,888) matched to controls (n=79,435) on age, sex, and year of encounter.

#### New York University School of Medicine

Investigators at NYU are examining the relationship between modifiable community factors and risk for T2D and obesity using a retrospective cohort assembled through EHR data from the Veterans Affairs Corporate Data Warehouse, a national repository of clinical and administrative data. The 2 primary exposures of interest are the food and housing environments. The assembled cohort includes more than 6 million veteran patients who were diabetes-free upon entry into the cohort from 2008 to 2016. Entry eligibility includes 2 primary care visits with no indication of diabetes within the 5 years before cohort entry, with at least two follow-up visits at least 30 days apart during the study period (2008-2018). The population has a well-documented high incidence of diabetes [36], providing adequate variation in contexts and outcomes to examine community factors in relation to T2D incidence.

For site-specific analyses, NYU’s primary community factors of interest are the food and housing environments. Food environment metrics include 2 absolute measures and 2 relative measures created from the RECVD data (Table 4). The NYU team also has store-level Nielsen Retail Scanner data from 2006 to 2014, which will be used to examine potential mechanistic pathways, including whether risks associated with living in select food environments are partially mediated through per capita sales of sugar-sweetened beverages. The NYU team is guiding Network decisions on the food environment measure development and harmonization, in collaboration with the DCC. They are also engaging in site-specific analyses to examine the influence of housing affordability per ACS and Veterans Affairs data on T2D risk.

NYU study outcomes include diabetes incidence and control as well as obesity prevalence and incidence. Outcome data are extracted from EHRs, capturing demographic, clinical, and utilization data. To ensure participants in the cohort do not have diabetes at cohort entry, individuals with any diabetes (type 1 or type 2) International Classification of Disease version 9 or 10 (ICD-9/10) code or elevated HbA_1c_ at enrollment are excluded. Time-to-event analyses (Cox proportional hazards models with frailty to account for clustering within a community) will be used to examine the main effects of the food environment on T2D risk and its role in mediating the association between NSEE and T2D risk. Person-time is calculated as the date of a censoring event (diabetes diagnosis, death, loss to follow-up, or end of study period) minus the date of cohort entry. The date of death is obtained from the Veterans Affairs Vital Status and Beneficiary Identification Records Locator. Loss-to-follow-up is defined as no Veterans Affairs encounter for more than 2 years but patients can re-enter the cohort if they meet entry criteria again.

#### University of Alabama at Birmingham

The UAB site is investigating the association of NSEE with a greater burden of T2D and cardiovascular risk, particularly in southeastern United States. To address site-specific and Network-wide research questions, UAB is leveraging resources from the REGARDS study [33]. The REGARDS study is a longitudinal, population-based closed cohort study of 30,239 adults aged 45 years and older at baseline (2003-2007), designed to identify factors associated with higher stroke mortality. The study was designed to oversample non-Hispanic Black adults and residents of the Stroke Belt region, with 56% of the sample selected from the Stroke Belt and the remaining 44% selected from the other 40 contiguous states. Demographics, medical history, and lifestyle factors were assessed at baseline and an in-home physical exam was performed with blood and urine collection. Follow-up is ongoing every 6 months to assess vital status and hospitalizations and obtain medical records for adjudication of possible cardiovascular events. A second in-home physical exam was completed between 2013 and 2016.

For site-specific analyses, the primary exposure includes the NSEE as assessed using principal component analysis for measures of community-level income or wealth, education, housing, health systems or services, employment, social environment, and physical environment. The data to assess these characteristics include both publicly available databases (eg, US Census) and commercial databases (eg, Dun & Bradstreet). The primary outcomes are incident T2D and cardiovascular outcomes. Incident T2D will be assessed among 11,199 REGARDS study participants without prevalent T2D at baseline and who completed the follow-up in-home physical exam during which objective measurements (eg, glucose, use of medications) were collected (Table 2). Cardiovascular outcomes include hypertension (ie, mean blood pressure140/90 mm Hg or use of hypertension medications) and expert adjudicated clinical events (ie, coronary heart disease, stroke).

Separate from the analysis of the REGARDS study data, UAB will utilize Medicare administrative claims data to investigate the association of NSEE with T2D and hypertension incidence. These data consist of several federal health care insurance programs that cover adults aged 65 years and older and younger individuals who are disabled or have end-stage renal disease. Broadly, Medicare Part A covers hospital services, Medicare Part B covers outpatient and physician services, and Medicare Part D covers prescription drugs. UAB will use the 5% random sample of Medicare claims data available from 1999 to 2015 to investigate community-level determinants of T2D incidence, diabetes hospitalizations, and treatment patterns. An overview of the Medicare sample population and diabetes definitions used for site-specific analyses is provided in Multimedia Appendix 2. Statistical approaches include generalized linear models and spatial generalized linear mixed models.

#### Drexel Data Coordinating Center

The Drexel DCC provides the study sites with project coordination and statistical support, including advanced methodological and analytic approaches to data analyses driven by the Network aims and heterogeneous data from each study site. The expertise needed for this work is reflected in the backgrounds of DCC team members, including biostatisticians and epidemiologists from the Dornsife School of Public Health and the Drexel Urban Health Collaborative (UHC), postdoctoral fellows, doctoral-level biostatistics students, data analysts and managers, and GIS experts. Through exploratory analytic work, including principal component analysis, exploratory factor analysis, GIS analysis and mapping, and correlation analysis of contextual indices against individual variables, the DCC supports the Network’s collective decision making around defining exposure metrics for addressing Network aims. The relationship with the UHC also allows for access to data from RECVD and other sources; provides support for GIS methods, data distribution, and storage; and provides access to data engineering experts. Furthermore, the UHC has a Policy and Outreach Core, which helps provide guidance on disseminating LEAD Network findings.

## Results

The Network has developed metrics for the community factors of interest: NSEE, food establishment, physical fitness establishment, leisure-time physical activity, and land use environments (Table 4). The Network has created these measures using data that are consistently available and contextually applicable to all geographies in the contiguous United States. This underscores the importance of the Network’s development of a method for categorizing community types for stratified evaluation of community factors with T2D onset. With harmonized measures, the Network is poised to compare findings across the varying study sites.

The Network has reported findings based on work from the initial years of funding. Preliminary results have been presented at annual meetings of the Society for Epidemiologic Research, the American Diabetes Association, and the American Public Health Association [52,53]. The Network recently published a paper describing county-level determinants of diabetes status in the United States from 2003 to 2012 [54]. The NYU team published a paper describing the impact of changes in the built and social environment on BMI in US counties using data from the Behavioral Risk Factor Surveillance System [13]. Additional manuscripts are in press or in development.

## Discussion

### Strengths and Limitations

The Diabetes LEAD Network leverages a breadth of expertise and data to advance knowledge regarding modifiable community risk factors for T2D onset and related outcomes. The Network brings strengths to its collective mission to provide scientific evidence for targeted interventions and policies. First, the sites provide the Network with community data sources that collectively ensure widespread geographic coverage and variation of community types across a rural-to-urban spectrum. It was of particular importance to ensure representation by rural communities, since CDC reports that diabetes is 17% more prevalent in rural than urban areas [55]. Furthermore, understanding the association between community and health requires the assessment of a heterogeneous set of communities [12,56]. Each of the study sites contributes unique data sources to achieve this goal. The NYU cohort spans the nation, the UAB REGARDS study cohort offers a national study with in-depth data from a high-risk region (Stroke Belt), and the G/JHU population is a regionally representative sample with high rural representation and primary data collection.

A second contribution of the Network is the development of measures of 6 community factors (Table 4) to be examined across diverse geographies and community types. These measures are being developed with consideration of community types, examining community factors within strata of different community types defined along a rural-urban spectrum to avoid potential differential item functioning and nonpositivity [23]. In addition, Network investigators are examining individual and joint associations to better understand how these community factors work in concert to contribute to excess T2D risk. With access to individuals’ residential and commercial addresses, the Network is evaluating spatial scales of various types and sizes to better understand the impact of scale on the findings.

Third, the range of expertise across institutions allows the Network to address methodological challenges common to community and health research [20]. In addition, the Network is advancing methods for conducting research on the role of community and health using data obtained from EHR systems, including extracting historical residential addresses to allow for time-varying exposure estimation. Finally, the Network is harmonizing community factor definitions and analytical approaches to facilitate comparable analyses and replicating analyses across 3 different study populations. This effort to harmonize approaches across multiple settings and populations will advance both the field of community and health research and generate data needed to guide evidence-based policies for T2D prevention in the United States.

There are some limitations to the Diabetes LEAD Network research portfolio. First, although access to longitudinal data will mitigate issues of temporality, the available data do not allow for investigation of early life exposures (eg, childhood) that may influence T2D risk [57]. Second, while the diversity of populations and geographies across the study sites is advantageous to expand the generalizability of findings and include previously underrepresented settings (ie, rural), it complicates comparison of results across sites. However, by harmonizing measurement and analytical approaches the Network will be well positioned to pinpoint reasons for any potential conflicting results that arise. Finally, despite employing advanced analytic approaches, the studies are all observational in design; thus, they are potentially constrained with respect to causal inference due to the risk of residual confounding and neighborhood self-selection [58,59]. The Network will consider methodological approaches such as propensity scores to address this limitation [43].

### Conclusions

T2D is a leading cause of morbidity in the United States, with select populations, often defined by geography, affected by a disproportionate burden of disease. The Diabetes LEAD Network identifies modifiable community factors that influence geographic disparities in T2D risk across diverse communities and identifies policy levers to ameliorate these disparities.

## Appendix

Multimedia Appendix 1 Description of primary data collection study for Geisinger and Johns Hopkins University site-specific analyses.

Multimedia Appendix 2 Description of Medicare study population for University of Alabama at Birmingham site-specific analyses.

## Abbreviations

ACS: American Community Survey
CDC: Centers for Disease Control and Prevention
CHD: coronary heart disease
CSD: community socioeconomic deprivation
DCC: data coordinating center
EDG: Epic Diagnostic code Groupers
EHR: electronic health record
GIS: geographic information systems
ICD: International Classification of Diseases.
LEAD: Location, Environmental Attributes, and Disparities
NETS: National Establishment Time Series
NSEE: neighbourhood socioeconomic environment
RECVD: Retail Environment and Cardiovascular Disease
REGARDS: Reasons for Geographic and Racial Differences in Stroke
RUCA: Rural-Urban Commuting Area
T2D: type 2 diabetes
UHC: Urban Health Collaborative
