# Supplementary material for: The Diabetes Location, Environmental Attributes, and Disparities Network: Protocol for Nested Case Control and Cohort Studies, Rationale, and Baseline Characteristics
Source: JMIR Res Protoc. 2020 Oct 19;9(10):e21377. doi: 10.2196/21377 (PMC7605983; doi:10.2196/21377)
Supplement: Multimedia Appendix 1 [file resprot_v9i10e21377_app1.docx]

Multimedia Appendix 1

| **Study population** | Inclusion criteria   - prevalent type 2 diabetes - age 18 years or older - at least two Geisinger clinical encounters between 2001 and 2016 with a primary care provider^a^ - Geisinger clinical encounter in the last two years - residential address in one of the 42 selected communities (described below)   Conducting data collection interviews with 475 individuals in each of 42 communities. Interviewing 325 participants a second time, for a total of 800 interviews. |
| --- | --- |
| **Study geography** | Inclusion criteria   - within the 37 county Geisinger service area - at least 125 individuals meeting population inclusion criteria (above)   Selected the communities with the lowest (n = 20) and highest (n = 20) mean HbA1c values |
| **Self-reported measures** | perceived stress scale  state-trait anxiety inventory  sleep PROMIS measure  food insecurity  medication adherence  social status ladder  transportation questionnaire  community perceptions  socioeconomic status: employment, income  residential history |
| **Biomarkers** | salivary and hair cortisol |
| **Anthropomorphic measures** | weight  height  waist circumference |
| **Direct measurement of communities** | physical activity promoting features  aesthetic quality  social cohesion to promote physical activity  barriers to physical activity  environmental features that promote safety  index of incivilities |
| ^a^Family medicine, internal medicine, pediatrics, gynecology/obstetrics. Abbreviations: HbA1c: glycated hemoglobin; PROMIS = Patient-Reported Outcomes Measurement Information System | |
