# Supplementary material for: The Diabetes Location, Environmental Attributes, and Disparities Network: Protocol for Nested Case Control and Cohort Studies, Rationale, and Baseline Characteristics
Source: JMIR Res Protoc. 2020 Oct 19;9(10):e21377. doi: 10.2196/21377 (PMC7605983; doi:10.2196/21377)
Supplement: Multimedia Appendix 2 [file resprot_v9i10e21377_app2.docx]

Multimedia Appendix 2

| **Aim** | **Spatial Scale for Contextual Domains** | **Study Years** | **Population Eligibility Criteria** | **Definitions** |
| --- | --- | --- | --- | --- |
| Assess community-level social determinants of health and the prevalence and incidence of diabetes in the US. | County and zip codes | 1999-2015 | - 5% random sample; - Age>=66 years; - Medicare Part A (in-hospital) and Part B (outpatient; institutional providers and carrier files) - Continuous coverage Part A + Part B, no HMO/Medicare Advantage | Primary diabetes based on 1 inpatient diagnostic code or 2 outpatient diagnostic codes at least 7 days apart.   - ICD-9 codes: 250.xx. - ICD-10 codes: E10, E11.   For type 2 diabetes-specific analyses, the codes for type 1 diabetes (e.g., E10, 250.x1, 250.x3) will be excluded.   - Index year with 2-year window will be used to assess type 2 diabetes incidence. |
| Assess pharmacologic treatment patterns and hospitalization rates by community-level social determinants of health. | County and zip codes | 2007-2015 | - 5% random sample; - Age>=66 years; - Medicare Part A (in-hospital), Part B (outpatient; institutional providers and carrier files), and Part D (pharmacy) - Continuous coverage Part A + Part B, no HMO/Medicare Advantage | Prescription drug event for diabetes medication. |
